# Relationship between psychosomatic complaints and circadian rhythm irregularity assessed by salivary levels of melatonin and growth hormone

**DOI:** 10.1186/1740-3391-9-9

**Published:** 2011-09-14

**Authors:** Mitsuo Nagane, Rie Suge, Shu-Ichi Watanabe

**Affiliations:** 1 Department of Educational Physiology, Faculty of Education, Chiba University, Japan; 2 Department of Physiology, Faculty of Medicine, Saitama Medical University, Japan

**Keywords:** chronotypes (morningness-eveningness), circadian rhythms, phase difference, healthy students, growth hormone, melatonin, psychosomatic complaints.

## Abstract

**Background:**

In university health care settings, students with psychosomatic complaints often have chronotypic problems. For this reason, we investigated a potential connection between psychosomatic complaints and circadian rhythm irregularity assessed by salivary levels of melatonin and growth hormone.

**Methods:**

Fifteen healthy students between 21 and 22 years of age were examined for physiological parameters of chronotypes based on melatonin and growth hormone secretion patterns, using a fluorescence enzyme immunoassay. Salivary samples were collected from subjects at home five times each day (20:00, 24:00, 04:00, 08:00, and 12:00 h). In addition, the subjects rated their psychosomatic symptoms twice (at 08:00 and 20:00 h).

**Results:**

A group with irregular circadian rhythm of melatonin (ICR) showed more psychosomatic complaints than a group with the regular circadian rhythm (RCR), especially for anxiety.

**Conclusion:**

Psychosomatic symptoms, particularly anxiety, may be associated with irregularity in melatonin and growth hormone rhythms, which can be altered by basic lifestyle habits even in healthy students.

## Background

Students suffering from psychosomatic complaints often have basic lifestyle problems such as short sleep duration [1] and nocturnal lifestyle [2]. An increasing number of human health problems are related to dysfunction or desynchrony of the circadian system [2-4]. Psychosomatic complaints refer to symptoms experienced by the individual with physical symptoms (e.g., head ache) and psychological symptoms (e.g., irritability). These psychosomatic symptoms, which are largely mediated by the autonomic nervous system, may be strongly influenced by an individual's lifestyle, and the current so-called "24-h society" may alter environmental conditions for students.

In a previous study [5], we found that psychosomatic symptoms may be associated with chronotypic dysfunction, as inferred from rhythmicity in growth hormone (GH) levels. The results indicated a relationship between self-assessment scores and salivary levels of GH: subjects with high self-assessment scores showed significant variability in GH secretion over the day, whereas subjects with low self-assessment scores did not. In the present study, we focused on circadian dysfunction and measured both salivary melatonin and GH in each subject to examine hormone secretion profiles, which may reflect a subject's circadian rhythms. Based on these profiles, we examined whether circadian dysfunction affects psychosomatic conditions as measured by a simple questionnaire. We sought to uncover an index of circadian rhythm modulation and examine the level of correspondence between hormonal data and an individual's self-rated psychosomatic symptoms.

The circadian pacemaker within the central nervous system regulates human sleep cycles, hormone secretion, subject alertness, objective performance levels, and other physiological functions over a 24-h period. Core body temperature, plasma cortisol, and plasma melatonin are three variables that are frequently used to estimate the phase of the human pacemaker rhythm [6]. The degree of a morning or evening chronotype is expressed as a score that correlates with the timing of an individual's sleep, wakefulness, temperature, melatonin, and cortisol rhythms [7,8]. Synchronization of endogenous circadian rhythms to the exogenous 24-h day is thought to be achieved primarily by light-induced effects on the circadian clock, as related to the subject's activity patterns. Light is the primary synchronizer of the human biological rhythm; different chronotypes should have different patterns of light exposure depending on individual lifestyles.

A recent study demonstrated the usefulness of salivary hormone analysis for GH levels [9]. Although evidence that melatonin plays a role in the regulation of GH secretion has been reported [10], the relationship between these hormones and daily lifestyle patterns remains poorly understood.

For assessing chronotypes, we used two important hormones, melatonin and GH, which show similar daily rhythmicity [5,11]. We assumed that the daily secretion pattern of melatonin is an important index for examining the effects of students' lifestyles on biological rhythms, because nocturnal melatonin secretion can be suppressed by exposure to light of several hundred luxes, e.g., ordinary room light [12], whereas GH is not [13]. As we showed previously [5], GH is associated with the self assessment of psychosomatic conditions, and GH insufficiency affects several psychological conditions, such as reduced vitality and energy, depressed mood, emotional lability, impaired self-control, anxiety, and increased social isolation in adults [14].

The purpose of this research was to explore the lifestyle of today's youth by utilizing the characteristics of melatonin and GH, both of which show circadian rhythms under naturalistic conditions. We measured secretion patterns of melatonin and GH as indices of a student's life rhythm and examined its effect on psychosomatic complaints.

## Methods

### Participants

Fifteen Japanese university healthy students (7 men and 8 women) ranging in age from 21 to 22 years of age and without major medical disorders participated in this study. The study design was approved by the Ethics Committee of Chiba University, Japan, and all subjects provided written informed consent. A self-assessment questionnaire concerning psychosomatic symptoms was developed in accordance with data from the Health Behaviour in School-Aged Children study of the WHO [15] and psychosomatic complaints scale for adolescents confirmed by factor analysis at assessment of validity and reliability of the scale [16]. The questionnaire for this study contained five items related to physical symptoms and five items pertaining to mental symptoms. The same questionnaire [5] was used to measure each individual's psychosomatic symptoms at home twice each day (08:00 and 20:00 h). The items were rated on a 4-point scale, with 1 = not true at all and 4 = completely true. The total score for the 10-item scale ranged from 10 to 40, with higher scores indicating a greater degree of psychosomatic complaints. Evening scores were compared with psychosomatic states in the morning. We assigned subjects to regular or irregular circadian rhythm groups (RCR and ICR groups, respectively) based on whether melatonin secretion was high until midnight or not [13].

### Sample Collection

Saliva was collected from the subjects' mouths into Salivette sampling tubes (Sarstedt, Germany) using polyester swabs, following 2 min of chewing. Samples were collected five times each day at home (20:00, 24:00, 04:00, 08:00, and 12:00 h). To measure the biological rhythms of the students' natural lifestyles, we did not control the timing of light exposure (e.g., lights on or off) across the circadian day. The day of sampling was required to be normal weekday (i.e., without special events, menstrual periods or stressful circumstances). They were instructed to keep usual diurnal rhythm (for example, meals, bed time and wake-up time) and to adopt their normal habit of awakening whether spontaneously or by alarm but to take their sample immediately upon awakening. During the sleep phase, subjects were instructed not to turn on the light. After sample collection, the saliva was stored at -20°C until use.

### Salivary hormone assay

The saliva samples were centrifuged at 3,000 rpm for 10 min to remove all mucin. A standard fluorescent immunoassay was used to assess the salivary melatonin and growth hormone concentrations in each sample. To avoid inter-assay variability, all determinations were performed in a single series. In the first step, a 96-well Costar plate (white polypropylene 3355; Corning, USA) was pre-coated with 100 μl of anti-melatonin (AB-T079; Advanced Targeting Systems, USA) and anti-growth hormone antibodies (2071800210; Quartett, Germany) and incubated for 1.5 h at room temperature. After incubation, the plate was washed three times with phosphate-buffered saline and blocked for 1 h. After washing, 100 μl of saliva was dispensed into each well and left for 1.5 h. After another washing, primary antibody (35514; Abcam, USA and FU47500254; Funakoshi, Japan) was added to the plates and incubated for 1.5 h. Incubation with a secondary antibody (NB120-7112; Novus Biologicals, USA) was then performed for 1 h. After washing, rabbit anti-ovine immunoglobulin (ECF Western blotting reagent pack; Amersham Biosciences, USA) was added. After 20 min of incubation, the plate was scanned using a Fluoromark microplate fluorometer (Bio-Rad, USA), with excitation at 485 nm and emission at 590 nm.

### Statistical analysis

A repeated-measures analysis of variance (ANOVA) was performed on the questionnaire scores [5], to compare 10 psychosomatic complaints across all subjects (n = 15) at 08:00 and 20:00 h. Two-way ANOVA (group by time) with repeated measures (five saliva samples) were calculated in the parameters' melatonin or GH increase and post hoc *t*-tests at each time point was performed to assess group differences. Next, Pearson's correlation coefficient was used to test the associations between melatonin and GH levels. The results were presented as means ± S.E.M. and the level of significance was set at .05.

## Results

### Melatonin and growth hormone secretion profiles

**Figure 1:**
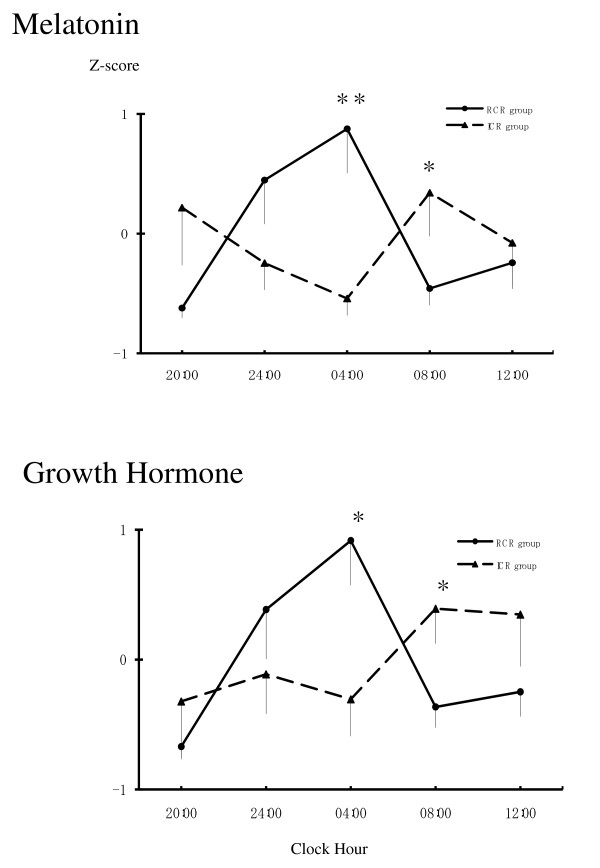
**Daily rhythms of melatonin and growth hormone secretion in the regular circadian rhythm (RCR, n = 8) group and irregular circadian rhythm (ICR, n = 7) group**. RCR group: there is a peak in melatonin and growth hormone secretion at 04:00 h. ICR group: there is no peak in melatonin and growth hormone secretion at 04:00 h. The vertical lines indicate standard errors of the means (SEM).

We collected saliva profiles from 15 healthy students (7 men and 8 women). The amplitudes, defined as the difference between the highest and lowest salivary concentrations, were determined for salivary melatonin and GH and were used to produce a standardization, or Z score. The subjects were assigned to regular (RCR; 3 men, 5 women) or irregular (ICR; 4 men, 3 women) circadian rhythm groups based on their melatonin rhythms. Figure 1 shows that, although large individual differences were present in both groups, melatonin and GH had begun to rise at 24:00 and peaked at 04:00 in the RCR group, whereas no peak in melatonin or in GH was observed at 24:00 h or 04:00 in the ICR group. Statistical analyses were performed using a correlation analysis and analysis of variance (ANOVA) for repeated measures. Melatonin rhythms were significantly correlated with GH levels in the RCR group (20:00 h, r = 0.90; 24:00 h, r = 0.98; 04:00 h, r = 0.99; 08:00 h, r = 0.89; 12:00 h, r = 0.93; with p < 0.01 for each); however, in the ICR group, the only significant correlation was at 08:00 h (r = 0.85, p < 0.05). Furthermore, although there were no differences in sleep duration between the two groups (RCR group, mean 6.10 h/day, mean bedtime 01:26 h; ICR group, mean 6.00 h/day, mean bedtime 02:51 h), sleep/wake timing was later in the ICR group.

As shown in Figure 1, the RCR group differed from the ICR group in terms of melatonin and GH secretion rhythms. Salivary melatonin and GH levels in the RCR group peaked at 04:00 h and reached a minimum at 08:00 h. In contrast, the levels of both hormones in the ICR group peaked at 08:00 h and were lowest at 04:00 h. Thus, the melatonin profiles differed significantly between the two groups at 04:00 (*t *= 3.38, df = 1, p < 0.01) and 08:00 h (*t *= -2.15, df = 1, p < 0.05), as did the GH profiles (*t *= 2.68, df = 1, p < 0.05 at 04:00 and *t *= -2.48, df = 1, p < 0.05, at 08:00 h).

### Psychosomatic symptoms profiles

**Figure 2:**
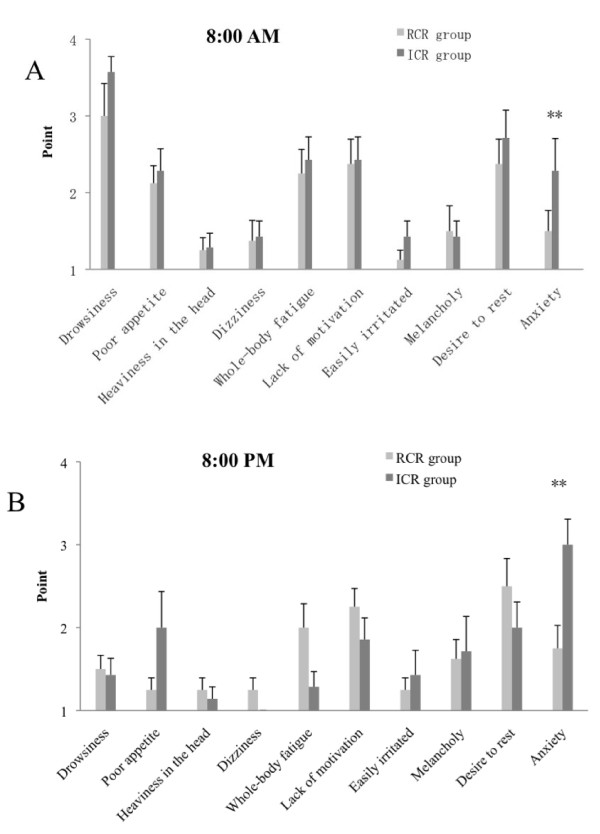
**Morning and evening psychosomatic condition scores in the regular circadian rhythm (RCR) and irregular circadian rhythm (ICR) groups**. The scores shown are means and SEM. Each scale ranges from 1 to 4, with a higher score indicating a greater degree of the psychosomatic symptom (No, Somewhat No, Somewhat Yes, Yes). (A) 8:00 AM; (B) 8:00 PM.

There was a main effect of time across all subjects (p < 0.01), but there was no significant group-by-time interaction with respect to psychosomatic symptom profiles. As shown in Figure 2A and 2B, psychosomatic complaints were high in the morning and decreased in the evening, especially for drowsiness (F = 40.95, df = 1/29, p < 0.01), poor appetite (F = 4.44, df = 1/29, p < 0.05), and whole-body fatigue (F = 5.20, df = 1/29, p < 0.05).

Next, an analysis between the RCR and ICR groups for each psychosomatic item was performed, revealing a significant difference in anxiety between the two groups at 08:00 and 20:00 h (Figure 2A and 2B), with the ICR group showing higher anxiety (F = 9.61, df = 1/29, p < 0.01). There were no significant differences in the other items between the two groups at the 08:00 or 20:00 h self-assessment.

## Discussion

### Physiological parameters based on melatonin and growth hormone rhythms

In this study, we devised a new physiological parameter for assigning chronotypes based on daily secretion patterns of melatonin as phase marker and GH. We divided the subjects into two groups, RCR and ICR, based on whether melatonin secretion was high until midnight. These two groups showed clear differences in daily secretion patterns (peak points) of GH and in the synchronization of the two hormones. As shown in the Results section, there are significant correlations between melatonin and GH secretion in the RCR group, but not in the ICR group (excluding 08:00 h). In contrast to the RCR group, the ICR group did not exhibit a characteristic profile for melatonin and GH, as there was no sharp peak typical of a normal profile [17,18]. Based on our recent results suggesting that GH and melatonin show similar secretion rhythms [11], we hypothesize that an individual's circadian rhythm can be more exactly determined by analyzing melatonin and GH as a pair. Therefore, we suggest that the asynchronicity and lack of peak secretion for the two hormones reflect an irregular circadian rhythm.

### Chronotypes and psychosomatic symptoms

It has been reported that morning chronotypes tend to perform well early in the day, while evening chronotypes show enhanced performance later in the day [19]. Some research reports that evening chronotypes go to bed later than midnight, but do not show signs of significantly short sleep duration because they also rise later in the morning. Furthermore, evening chronotypes omit breakfast, but consume adequate energy because they eat much more at night [17].

Evening chronotypes report psychological and psychosomatic disturbances more frequently and intensively than morning chronotypes, who tend to have a healthier lifestyle [20]. In our research during normal weekday under naturalistic conditions, the ICR group tended to more frequently complain of negative psychosomatic conditions, including feeling anxious (p < 0.01 compared with RCR).

In the present study, the circadian rhythm profiles of salivary melatonin and GH in the ICR group exhibited broad peaks that shifted towards the morning, as compared with the RCR group. It has been reported that peak hormonal secretion often shifts to the morning when an activity continues long into the night [13,21]. We hypothesize that psychosomatic states in the morning are deeply reflective of hormonal secretion. Therefore, morning psychosomatic state may be associated with lifestyle. That is, we hypothesize this psychosomatic disturbances fundamentally originate in phase and amplitude differences of circadian rhythm.

### Chronotypes and anxiety

Assessments of psychosomatic health complaints in European students show that the highest rates of complaints are found for physical states such as backache, headache, and neck ache [22], in addition to nervousness and irritability [15]. However, a survey of Japanese high school students indicates that psychological items related to attentiveness, depressive state, and irritability are more often affected. For chronotypes in Japanese students, increases in psychological stresses [16] related to anxiety are likely to have multiple causes.

It has been suggested that Japanese students suffering from psychosomatic disorders such as those involving mood and sleep may exhibit basic lifestyle problems, including deleterious changes in their living environment and dietary or lifestyle disturbances [2]. In particular, staying up late is associated with decreased appetite and missed breakfast the following morning, irregular bowel movements, and sleepiness. In our research, physical complaints such as those observed in European students were rather low, and differences between our groups were only observed for anxiety. Assuming that the ICR group reflects problems due to irregular lifestyle, our results are consistent with differences observed between European and Japanese students with respect to psychosomatic complaints reported thus far.

Our results indicate a relationship between melatonin and GH levels and self-assessment scores, exclusively anxiety. In contrast to subjects in the RCR group, subjects in the ICR group tended to feel anxiety not only in the morning but also in the evening, although both groups had high anxiety scores. Anxiety in Japanese youth related to basic lifestyle habits may be associated with hormonal rhythms. The present research revealed that nocturnal lifestyle in students increased their risk of psychosomatic health problems, including anxiety.

### Limitations

In this study, we attempted to clarify the association between psychosomatic symptoms and melatonin and GH levels; however, our study has limitations. First, the number of subjects studies was relatively small. Second, the sleep-disrupting effect of waking at 24.00 h and 04.00 h to produce a saliva sample might have influenced hormone secretion. Third, in spite of our attempt to avoid menstrual cycle problems, gender differences may have influenced the results.

## Conclusion

Our results suggest that psychosomatic symptoms, particularly anxiety, may be associated with melatonin and growth hormone rhythms, which are a direct result of basic lifestyle habits, even in healthy students. We propose that physiological analyses of a student's circadian rhythm should include measurements of melatonin and GH secretion as phase markers. We believe it is important to focus our attention on student's basic lifestyle problems, as an irregular biological rhythm may increase the prevalence of psychosomatic complaints.

## Competing interests

The authors report no conflicts of interest. The authors alone are responsible for the content and writing of this paper.

## Authors' contributions

MN designed the experiments, collected data and wrote the manuscript. RS participated in the design of this study and analyzed data. SW managed the laboratory and participated in the analysis and discussion of the results. All authors read and approved the manuscript.
